# Clinical profiling and medical management of Israeli individuals with Phelan McDermid syndrome

**DOI:** 10.1186/s13023-025-03598-3

**Published:** 2025-03-18

**Authors:** Odelia Chorin, Lior Greenbaum, Shelly Lev-Hochberg, Neta Feinstein-Goren, Aviva Eliyahu, Hagit Shani, Elon Pras, Tal Weissbach, Yoav Bolkier, Gali Heimer, Dorit Lev, Marina Michelson, Miriam Regev, Sagi Josefsberg, Nurit Assia Batzir, Adel Shalata, Ronen Spiegel, Reeval Segel, Orit Lobel, Bassam Abu-Libdeh, Mordechai Shohat, Moshe Frydman, Ronen Hady-Cohen, Ben Pode-Shakked, Annick Rein-Rothschild

**Affiliations:** 1 Institute of Rare Diseases, Edmond and Lily Safra Hospital for Children, Sheba Medical Center, Tel Hashomer, Ramat Gan, Israel; 2 The Danek Gertner Institute of Genetics, Sheba Medical Center, Tel Hashomer, Ramat Gan, Israel; 3 Faculty of Medical and Health Sciences, Tel Aviv University, Tel Aviv, Ramat Gan, Israel; 4 Department of Obstetrics and Gynecology, Sheba Medical Center, Tel-Hashomer, Ramat Gan, Israel; 5 Pediatric Cardiology Unit, Edmond and Lily Safra Hospital for Children, Sheba Medical Center, Tel Hashomer, Ramat Gan, Israel; 6 Pediatric Neurology Unit, Edmond and Lily Safra Hospital for Children, Sheba Medical Center, Tel Hashomer, Ramat Gan, Israel; 7 Magen Center for Rare Diseases, Wolfson Medical Center, Holon, Israel; 8 The Genetics Institute, Kaplan Medical Center, Rehovot, Israel; 9 Pediatrics Genetics Unit, Schneider Children’s Medical Center of Israel, Petach Tikvah, Israel; 10 Genetics Institute, Bnai Zion Medical Center, Haifa, Israel; 11 Ruth and Bruce Rappaport Faculty of Medicine, Technion-Israel Institute of Technology, Haifa, Israel; 12 Department of Pediatrics B, Metabolic Service, Emek Medical Center, Afula, Israel; 13 Institute for Rare Diseases, Emek Medical Center, Afula, Israel; 14 Shaare Zedek Medical Center, Wolf Children‘s Hospital, Jerusalem, Israel; 15 Faculty of Medicine, Hebrew University of Jerusalem, Jerusalem, Israel; 16 Department of Pediatrics, Makassed Hospital and Faculty of Medicine, Al-Quds University, East Jerusalem, Israel; 17 Maccabi Genetic Institute, Maccabi Health Services, Tel Aviv-Yafo, Israel; 18 Bioinformatics Unit, Sheba Cancer Research Center, Ramat Gan, Israel

**Keywords:** *SHANK3*, Phelan–McDermid syndrome, Autism spectrum disorder, Intellectual disability, Copy number variant, Single nucleotide variant

## Abstract

**Background:**

Phelan–McDermid syndrome (PMS) is a neurodevelopmental disorder, caused by haploinsufficiency of the *SHANK3* gene. In addition to global developmental delay (GDD)/intellectual disability (ID) and autism spectrum disorder (ASD), PMS is characterized by multiple neurologic, behavioral and multisystemic manifestations.

**Methods:**

We aimed to establish a database of individuals with PMS in Israel. All participants underwent a detailed evaluation at a single medical center, and demographic, clinical, and genetic data were collected.

**Results:**

Seventeen unrelated individuals with PMS (mean age 10 ± 8.2 years; range, 2.5–36 years) were enrolled (10 females, 59%), all of Jewish descent. Twelve cases (70%) were caused by deletions in chromosomal region 22q13.3, including mosaicism, ring chromosome and unbalanced translocation. The other 5 (30%) cases were due to single nucleotide variants (SNVs), while the de novo SNV c.3904dup (p.Ala1302GlyfsTer69), recurred in 3 cases. All 17 participants had GDD/ID (which was severe in 10, 59%), and ASD and seizures were present in 12 (70%) and 8 (47%) individuals, respectively. Additional frequent manifestations were sleep difficulties in 13 individuals (76%), bowel movement disorders in 13 (76%), urinary track involvement in 8 (47%) and endocrine disorders in 6 (35%). Abnormal but nonspecific findings on prenatal ultrasonography were noted in 3 participants (18%). The most common perinatal complication was prolonged jaundice in 5 infants (29%). Different medical treatment modalities, including cannabidiol (CBD) full-spectrum oil extracts, were used to ease symptoms, with variable results.

**Conclusions:**

Our experience adds to current knowledge about clinical manifestations and potential symptomatic treatment of PMS in Israel. These findings may promote clinical research and serve as infrastructure for future clinical trials.

## Background

Phelan McDermid Syndrome (PMS, OMIM #606232) is a rare autosomal dominant neurodevelopmental disorder characterized by developmental delay (DD)/intellectual disability (ID), autism spectrum disorder (ASD), epilepsy, behavioral abnormalities, and other systemic features [1–3]. PMS is caused by haploinsufficiency of the *SHANK3* gene (located on chromosomal region 22q13.3), involving deletion of the telomeric region encompassing *SHANK3* (in approximately 75% of individuals) or pathogenic single nucleotide variants (SNVs) within this gene (in a minority of cases). However, additional complex chromosomal rearrangements have been reported [4, 5]. While most cases are attributed to de novo variants, rare cases of an inherited variant with reduced penetrance and variable clinical expressivity have been documented [4, 6, 7]. *SHANK3* encodes a protein involved in the regulation of the postsynaptic density of glutamatergic synapses and is pivotal in the regulation of synaptic function and dendritic formation [2, 8, 9].

In addition to neurodevelopmental features, the multisystem manifestations of PMS include dysmorphic facial features, reduced pain perception, hearing and visual abnormalities, lymphedema, and gastrointestinal and endocrine symptoms [5–7]. Multiple congenital malformations, such as cardiac and renal anomalies, have been described [2, 7]. Variable brain magnetic resonance imaging (MRI) findings are recognized, including cerebellar vermis hypoplasia and corpus callosum abnormalities, whereas some individuals present with normal imaging [10–12].

Previous studies reported prenatal ultrasonographic findings in fetuses affected by PMS, including increased nuchal translucency and intrauterine growth restriction (IUGR), along with additional variable cardiac, gastrointestinal, renal, or skeletal malformations [1, 13–17].

More than 1500 participants have been registered in the PMS International Registry. Nevertheless, the exact prevalence of the disease remains unknown, most likely due to underdiagnosis [18]. PMS has been estimated in some studies to account for 0.5-2% of cases of ASD and ID [19–20]. Thus far, there are no specific therapeutic options for this syndrome, and care is aimed at improving neurobehavioral outcomes and quality of life.

We describe herein a cohort of PMS individuals from Israel, all of whom were evaluated at the Institute for Rare Diseases (IRD) in a large tertiary pediatric hospital at Sheba Medical Center (SMC). The systematic data collection of PMS epidemiology, natural history, and genotype‒phenotype correlations are important for optimizing patient care. Furthermore, it may assist in translational research for future therapeutic agents, as well as in the recruitment of participants for clinical trials, when eligible.

## Materials and methods

### Participant recruitment and evaluation

To establish a comprehensive database of PMS in Israel, two main routes of recruitment were used. First, individuals were referred by the Israeli PMS parents’ advocacy group and by social media advertisement. Families were also invited to attend a half-day meeting dedicated to PMS, which was conducted at SMC. Second, the IRD team approached medical professionals from across Israel and asked them to refer patients diagnosed with PMS.

All PMS individuals were invited for consultation at the IRD and examined by a single pediatrician and medical geneticist (OC). Demographic and clinical characteristics, data about natural history, and results of genetic testing were obtained from parents, family members and/or caregivers via a structured phenotyping questionnaire composed specifically for this purpose. Participants were particularly asked about medications, brain imaging findings and prenatal ultrasonographic surveillance. Notably, we did not revise the imaging data and relied on medical reports. We discussed with the families the diagnosis, treatment options, recommendations regarding medical surveillance and risk of PMS recurrence in future pregnancies of family members. Follow-up consultations in our clinic were provided according to request/need.

### Molecular diagnosis

Molecular diagnosis was achieved via chromosomal microarray analysis (CMA) (*n* = 10), comparative genomic hybridization (CGH) (*n* = 1) or clinical exome sequencing (ES) (*n* = 6). We revised the classification of all copy number variants (CNVs) and single nucleotide variants (SNVs) according to the American College of Medical Genetics and Genomics (ACMG) guidelines [21, 22]. Only individuals harboring pathogenic and likely pathogenic variants encompassing *SHANK3* were included in the cohort.

Additional genetic testing, including karyotyping and fluorescence in situ hybridization (FISH) for individuals and parents, was conducted when indicated.

## Results

### Demographic characteristics and molecular diagnosis

Between 2019 and 2023, 17 individuals with PMS (age at visit: mean 10.1 ± 8.2 years, range 2.5–36 years) attended our clinic and included in this cohort, 10 of whom (59%) were females and 7 were males (41%). Two were adults, aged 20 and 36 years. The participants were from 17 unrelated families of Jewish descent. The mean age at molecular diagnosis was 6.4 years ± 6 years (range 8 months-27 years), and evaluation was performed at 8 genetic institutes (Table 1).

As presented in Table 2, 12 participants (70%) harbored chromosomal deletions encompassing the 22q13.3 region. Two presented with mosaicism for ring chromosome 22, one of whom also displayed mosaicism for monosomy 22 (in 10% of cells). One individual presented with mosaicism for the 22q13.3 terminal deletion (in 20% of the cells).

**Table 1:** Demographic and clinical characteristics of individuals diagnosed with Phelan-McDermid syndrome

Participant #	Sex	Age at evaluation (years)	DQ	ASD	Developmental regression	Verbal communication	Age at independent walking (months)	Neuropsychiatric comorbidities	Brain MRI	Seizures
1	F	5.5	< 50	N	Y	N	19	ADHD	Normal	Y
2	F	2.5	< 50	Y	Y	N	20	N	Normal	Y
3	M	9	< 50	Y	Y	N	16	ADHD	Normal	N
4	F	3.5	< 50	Y	N	N	Non ambulatory	N	Thin CC, pineal cyst, arachnoid cyst with sylvian fissure enlargement	N
5	F	13	< 50	Y	Y	N	30	Behavioral outbursts	Mild enlargement with dysplastic lateral ventricles, thin CC	Y
6	M	10	N/A	N	N	Y Speech dyspraxia	14	Anxiety, behavioral outbursts	Normal	Y
7	F	4.5	< 50	N	Y	Y Speech dyspraxia	Non ambulatory	N	WM abnormalities	N
8	M	36	< 50	Y	Y	N	12	ADHD	Ventriculomegaly	N
9	F	12	< 50	Y	Y	Y	36	N	Normal	Y
10	F	6	76	Y	Y	Y	20	N	N/A	N
11	F	7	< 50	N	Y	N	26	ADHD	WM abnormalities	N
12	F	20	N/A	Y	Y	N	24	Behavioral outbursts	Parietal lobe atrophy and WM abnormalities	Y
13	M	12	70	Y	N	Y	18	ADHD, OCD	N/A	N
14	M	6	55	N	Y	Y	20	ADHD	N/A	N
15	M	3.5	60	Y	N	Y	15	ADHD	Susp old hemorrhage in cerebellum	Y
16	F	17	< 50	Y	Y	N	14	Anxiety, depression, tics	Normal	Y
17	M	4.5	85	Y	N	Y	12	N	N/A	N

Abbreviations: ADHD – attention deficit hyperactivity disorder, ASD- autism spectrum disorder, CC – Corpus Callosum, DQ – developmental quotient, F-female, M-male, MRI – magnetic resonance imaging, N- no, N/A – not available, OCD – obsessive‒compulsive disorder, Y-yes

**Table 2:** Results of molecular diagnosis and parental testing

Participant	CMA	Karyotype	FISH	Exome sequencing	Parental testing	Age at molecular diagnosis
1	Mosaic for 7.3 Mb deletion 22q13.2q13.33 × 1	46,XX (80%), 46 XX del(22)(q13.3)(20%)	N/A	N/A	N/A	1.5
2	Benign	N/A	N/A	Chr22:51159932 T > TG, c.3904dup, p.Ala1302GlyfsTer69 (RCV000004730.21)	DNV (*)	4.5
3	Benign	N/A	N/A	Chr22-51160683 G > A, c.4608G > A, p.Trp1536Ter (RCV002214268)	DNV (*)	5.5
4	7.2 Mb deletion 22q13.2-q13.33 (chr22:43980615–51197766)x1; 578.8Kb deletion 22q11.1 (16923916–17502729)x1	46,XX, del(22)(q13.3)	* ish del 22q13,3, ish inv22 q11.2	Benign	karyotype + FISH, WES- DNV	0.5
5	6.7 Mb deletion 22q13.31-22 (22:44507878–51304566)*1 1 Mb duplication 10p15.3 (0-1066497)*3	N/A	N/A	N/A	karyotype + FISH- paternal translocation t(10;22)(p15.3;q13.3)	8
6	185Kb deletion 22q13.33 (51,013,156 − 51,197,766)*1 452Kb duplication 5q35.3 (180,262,937 − 180,715,096)*3	N/A	recommended- NA	N/A	N/A	7
7	3.77 Mb deletion 22q13.3-q13.33 (22:47534670–51304566)x1	46,XX, (50%), 45,XXr22 (40%), 45,XXdel22 (10%)	* ish mosaic (40%) del 22q13,3	N/A	karyotype + FISH normal	1
8	Benign	N/A	N/A	Chr22:51159932 T > TG, c.3904dup, p.Ala1302GlyfsTer69 (RCV000004730.21)	DNV (*)	27
9	Mosaic deletion22q13.2-q13.3 (40%), (47664055–51178235)*1	46,XX/ 45,XXr22	* ish mosaic (40%) del 22q13,3	N/A	CMA + FISH- normal	2.5
10	954 Kb deletion 22q13.33 (50,350,557 − 51,304,566)*1	N/A	N/A	N/A	FISH- normal	2.0
11	8.2 Mb deletion 22q13.2-q13.3 (43007972–51197838)*1	46,XX, del(22) (q13.3)	* ish del 22q13,3	N/A	FISH- normal	1
12	4.3 Mb deletion22q13.2**	46,XX	* ish del 22q13,3	N/A	FISH- normal	9
13	Benign	N/A	N/A	Del: Chr 22:51154080-51179125-Hg19 (25Kb from 22q13.2-13.3)	DNV (*)	9.5
14	744 Kb deletion, 22q13.33 (50560618–51304566)*1	46XY	N/A	N/A	FISH- normal	5
15	55Kb deletion 22q13.3 (51122452–51178264)*1	46XY	* ish del 22q13,3	Del: chr22:51123003–51,216,394 (93.4Kb deletion from 22q13.3)	FISH- normal	2.5
16	Benign	N/A	N/A	Chr22:51159932 T > TG, c.3904dup, p.Ala1302GlyfsTer69 (RCV000004730.21)		15
17	Benign	N/A	N/A	chr22:51133473 TC > T, c.1527delC, p.L510SfsTer32	Paternally inherited variant (DNV in father)	4.5

* WES- Confirmed parental status

**CGH

Abbreviations: CMA, chromosomal microarray analysis; DNV, de novo variant; FISH, fluorescence in situ hybridization; Kb, kilobase; Mb, megabase; N/A, not available; WES, whole-exome sequencing

CMA results are in accordance with GRCh37/hg19, and SNVs refer to NM_001372044.2

**Fig. 1:**
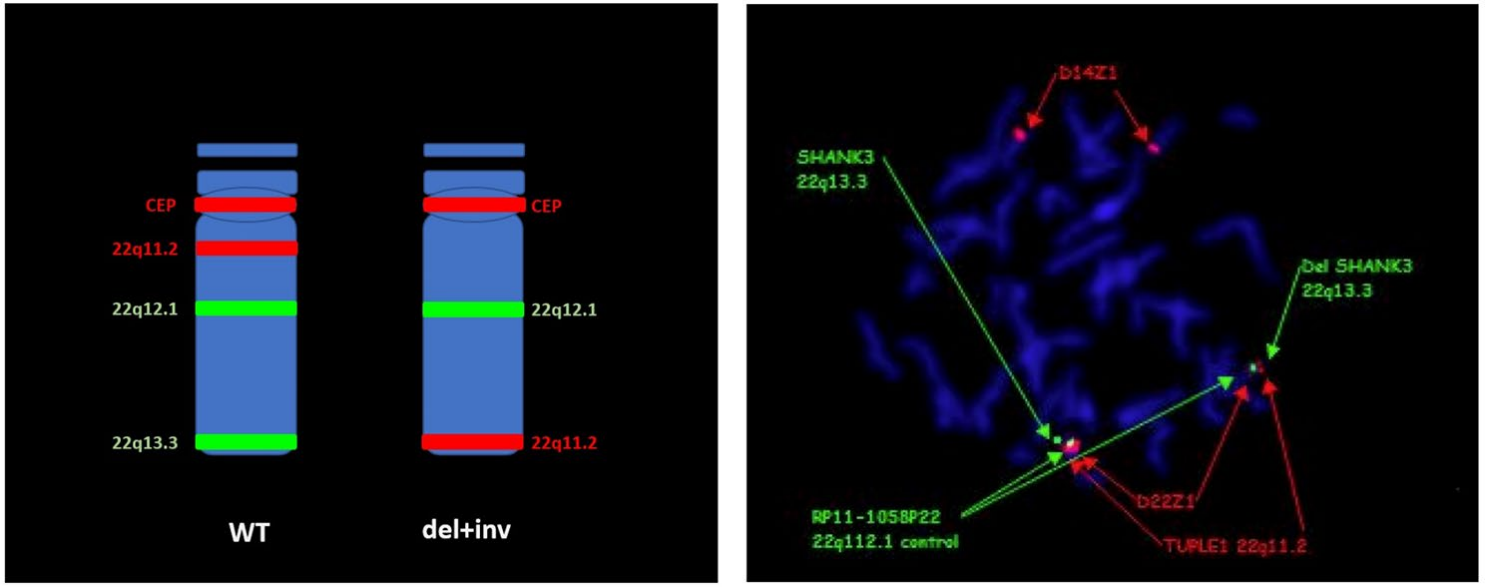
FISH markers on chromosome 22. CEP = D22Z1 (red); 22q11.2 = TUPLE1 D22S451 (red); 22q12.1 = RP11-1058P22 (green); 22q13.3 = SHANK3 D22S1254. WT- wild type

Additionally, one individual harbored two chromosomal deletions within the long arm of chromosome 22: 579Kb in 22q11.1 (16,923,916 − 17,502,729, Hg19) and 7.2 Mb in 22q13.3 (43,980,615 − 51,197,766), raising suspicion for a complex chromosomal rearrangement. Initial FISH testing revealed a distal deletion. Consequent testing with additional region-specific probes revealed an inversion leading to a unique chromosomal rearrangement (Fig. 1). 

In another individual, PMS was attributed to an unbalanced translocation due to a parental balanced translocation (t(10;22)(p15.3;q13.31)). In this family, a sister of the parent harboring the translocation was reported to have a clinical phenotype that coincides with PMS. However, the family declined further genetic testing. A cousin presenting with developmental delay and facial dysmorphism had a mirror chromosomal aberration with a duplication of 22q13.3 and deletion of 10p15.3.

In total, CNVs were de novo in 11 individuals, while one was secondary to balanced parental translocation, as mentioned previously. However, in one individual, the complex structural abnormality of an inversion and deletion may suggest an undetected parental chromosomal abnormality. In one case, the deletion size (~ 25 kb) was below the CMA resolution threshold for detection and was determined via next-generation sequencing (NGS).

In 5 individuals, PMS was caused by a SNV, all of whom were diagnosed as part of trio ES, as presented in Table 2. Four of the variants were de novo (Table 2). The de novo SNV NM_001372044.2:c.3904dup, p.Ala1302GlyfsTer69, recurred in 3 individuals. This variant was previously reported and named c.3679dup; p.Ala1227Glyfs*69 [23]. A single variant, NM_001372044.2:c.1527del, p.L510Sfs*32, was not previously reported (participant 17, Table 2) and was paternally inherited. Typical features of PMS were absent in the father; however, he was diagnosed with attention deficit/hyperactivity disorder (ADHD). Familial segregation within this family revealed the variant to be de novo in the proband’s father and absent in the proband’s healthy sibling. Notably, descriptions of individuals 2 and 3 (Table 2) were previously published [24, 25].

### Developmental, neurological, and behavioral phenotypes

**Fig. 2:**
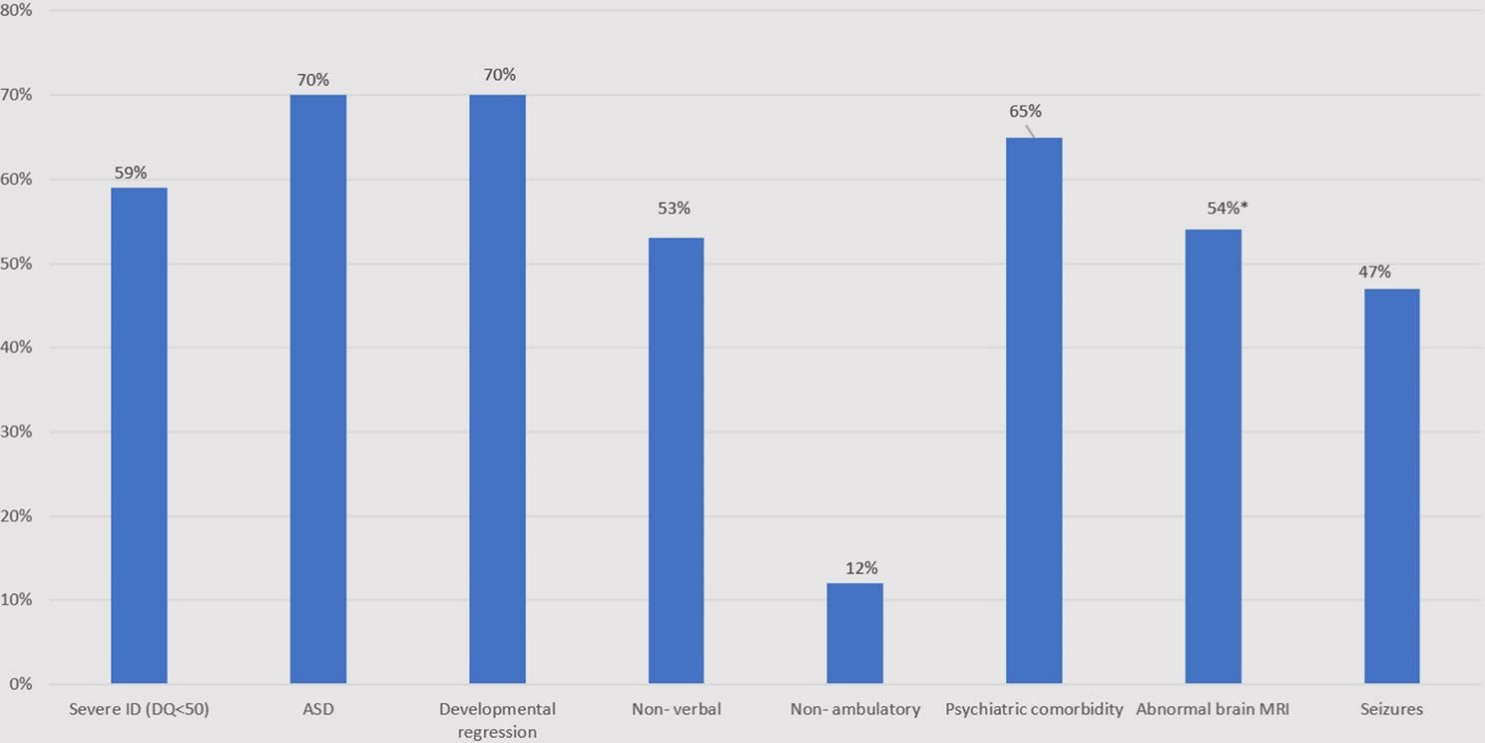
Neurodevelopmental characteristics of individuals with Phelan-McDermid syndrome. ASD- autism spectrum disorder; DQ- developmental quotient; IQ- intellectual quotient; MRI- Magnetic resonance imaging; PMS- Phelan McDermid syndrome. * 7/ 13 individuals who underwent MRI

All 17 participants presented with neurodevelopmental phenotypes, including ID, which was classified as severe (developmental quotient (DQ) < 50) among 10 (59%) individuals. Twelve individuals were diagnosed with ASD (70%). Regression was reported in 12 participants (70%), with loss of acquired verbal (*n* = 4), motor (*n* = 1), or verbal and motor (*n* = 7) skills. In 10 cases, the regression was reported between ages 1–7 years, and in two other cases multiple episodes of regression occurred from childhood into adolescence with consequent mild improvements. The mean age of ambulation attainment was 20 ± 7 months (range 12–36 months), whereas 2 individuals remained non ambulatory (aged 3.5 and 4.5 years at last evaluation). Eight participants (47%) attained verbal skills at last evaluation, two of whom were diagnosed with speech dyspraxia (Fig. 2). Alternative communication techniques, including Grid, typing, cards and iPads, were used by 6 individuals (35%).

Seven participants (41%) were diagnosed with epilepsy, with generalized tonic‒clonic the most common type of seizures (*n* = 6), whereas febrile seizures were reported in one individual and a suspected neonatal seizure at 48 h of age in another neonate with a normal EEG and without recurrence. In all but one individual, seizures resolved or were well controlled under a single drug regimen (e.g., valproic acid, barbiturates, or medical cannabis).

Increased pain tolerance was reported in all but one individual (94%), with decreased perspiration or heat intolerance reported among 10 participants (59%).

The behavioral and psychiatric comorbidities were ADHD (*n* = 7, 41%), agitation and restlessness (*n* = 2, 12%), anxiety (*n* = 1, 6%), obsessive‒compulsive disorder (OCD) (*n* = 1, 6%) and other behavioral abnormalities (*n* = 3, 18%). Treatment modalities included atypical antipsychotics (*n* = 3, discontinued in 2), phenothiazines (*n* = 3, all discontinued treatment), stimulants (*n* = 3, discontinued in 2), serotonin-specific reuptake inhibitors (SSRIs) (*n* = 1, discontinued), and serotonin norepinephrine reuptake inhibitors (SNRIs) (*n* = 1). Reasons for discontinuation of treatments included behavioral changes and lack of improvement.

One individual received a treatment trial of insulin-like growth factor 1 (IGF1) and oxytocin as part of compassionate care, both of which were discontinued due to a lack of improvement in symptoms. Additional data regarding the regimen and duration of this treatment are lacking. Another individual with hypotonia was treated with amantadine (ages 2–4 years), with reported improvement in muscle tone. The treatment was discontinued after two years due to symptom resolution.

Sleep difficulties, such as premature arousal and multiple night arousals, were reported in 13 individuals (76%). Therapeutic agents included antihistamines (*n* = 2, discontinued in 1), triclonam (*n* = 2, discontinued in both), benzodiazepines (*n* = 3) and melatonin (*n* = 6, discontinued in 2), whereas lack of improvement prompted discontinuation of treatment.

Brain imaging (MRI) was conducted in 13 participants (76%) and reported normal in 6 individuals. In the remaining 7 individuals, abnormal findings were observed in the white matter (*n* = 4), corpus callosum (*n* = 2), ventricular volume (*n* = 2), and/ or other findings (Table 1).

Eight participants (47%) were treated with cannabidiol (CBD) full-spectrum oil extracts. All individuals were treated with T1C20 products (drop content: 6.4 mg CBD & 0.32 mg THC) from two commercial brands. The indications for treatment were antiseizure medication and mood regulation. In one individual, treatment was discontinued after 3 months. The other 7 individuals were still being treated at last clinical evaluation, with a treatment duration of 9–34 months thus far.

### Growth parameters and dysmorphic facial features

All participants were born at term, with a mean birth weight of 3.3 kg (median 3.2 kg, range 2.6–4.3 kg). At last clinical evaluation, the mean length/height was at the 36th percentile for age (WHO charts, median 35.5%ile, range 0.4–97%ile), the weight at the 42nd percentile (median 50th %ile, range 70–80%ile) and the mean head circumference at the 40th percentile (median 42nd %ile, range 0.4–84%ile).

Variable mild facial dysmorphisms, as previously described in PMS, were notable in 10 individuals (59%). These included widow’s peak, frontal bossing, synophrys, bulbous nose, long philtrum, thin upper lip, high arched palate, retrognathia and pointed chin.

### Multisystem manifestations

Conductive hearing loss was documented in 7 participants (41%, bilateral *n* = 6, unilateral *n* = 1), which resolved following VT tube insertion. Four individuals (23%) had ophthalmologic abnormalities: strabismus (*n* = 2), astigmatism (*n* = 2) and/or lacrimal duct obstruction (*n* = 1).

Seven individuals underwent echocardiography (41%), which was normal in five, while atrial septal defect (ASD) and patent foramen ovale (PFO) were documented in a single individual each. One participant had a dual diagnosis of PMS and long QT (QTc 0.47 msec), secondary to a likely pathogenic de novo variant in the *KCNQ1* gene (NM_000218.3: c.658 C > T, p.Gln220Ter).

Eight participants achieved urinary and fecal continence (47%), two of whom had nocturnal enuresis. The latest reported age for acquiring toilet training was 7 years. Feeding difficulties ranged from failure to thrive in infancy (*n* = 1) to increased appetite (*n* = 3). Bowel movement disorders such as constipation, diarrhea and recurrent vomiting were reported in most participants (*n* = 13, 76%). Pica was noted among 3 (18%), whereas two individuals suffered from lactose intolerance, and one was diagnosed with celiac enteropathy. Dental anomalies, including multiple dental caries (*n* = 5) and bruxism (*n* = 5), were documented in 7 participants (41%).

Eight individuals (47%) presented with urinary tract involvement, among them recurrent urinary tract infections (UTIs) (*n* = 5) and hydronephrosis ranging from mild (*n* = 3) to severe (*n* = 1), accompanied by enlarged kidneys (*n* = 1) and hydroureters (*n* = 2).

Orthopedic abnormalities were documented in 5 participants (29%): hyperlaxity of joints, torticollis, scoliosis, kyphosis, bilateral forefoot adduction, and developmental dislocation of the hip (DDH). Lymphedema was observed in 4 individuals (23%).

Endocrine abnormalities were reported among 6 individuals (35%) and included precocious puberty (*n* = 3), premature adrenarche (*n* = 2) and growth hormone deficiency (*n* = 1).

### Prenatal and perinatal findings

All participants were queried regarding prenatal ultrasonography surveillance. Abnormal prenatal findings were documented in 3 (18%), with mild bilateral hydronephrosis in 2 individuals, along with a corpus callosum cyst in one. The third individual presented with microcephaly, which was initially noted during the third trimester.

Perinatal complications were jaundice in 5 individuals (29%), two of whom required prolonged (> 48 h) phototherapy. Data regarding hemolytic anemia risk factors are lacking.

## Discussion

The data from this cohort of Israeli PMS individuals, in line with previous reports from other cohorts, underscores the wide phenotypic variability of this condition, which includes neurodevelopmental and variable multisystem manifestations. To the best of our knowledge, this is the first cohort of PMS reported in Israel. While its size is relatively small, several important insights may be gained from our experience, mainly concerning the variety of CNVs that cause PMS and options for symptomatic treatment.

We did not observe major phenotypic differences between individuals with CNVs and individuals with SNVs or the impact of CNV size on phenotypic variability. Levy and colleagues stratified PMS individuals into 3 classes based on CNV length and gene content (class I, *SHANK3* +/- *ARSA* +/- *ACR* +/- *RABL2B* genes; class II, all other deletions) or sequence variants (class III) [26], however all participants with CNVs in our cohort had class II deletions. Surprisingly, two individuals (participants 1&9) with mosaicism for chromosomal deletions presented with a severe neurodevelopmental phenotype (DQ < 50), in line with the clinical features of individuals without mosaicism. This phenomenon has previously been reported by Phelan et al., in an individual with 8% mosaicism for ring chromosome 22 and typical features of PMS [7]. This may be due to a higher level of mosaicism in the brain, along with the contribution of additional, currently unrecognized, genetic and/or environmental factors.

Unique chromosomal rearrangements, including parental chromosomal rearrangements, inversions, and ring chromosomes, have previously been reported in PMS [27–28]. While CMA is often used as a first-tier test for individuals with ID/ASD, the telomeric deletion encompassing *SHANK3* region prompts further testing via cytogenetic methods. According to recent guidelines [29], karyotype and FISH in the probands are required to assess for unbalanced rearrangements with consequent parental testing as appropriate. In our cohort, 3 individuals harbored 22q13.3 deletion along with an additional CNV (individuals 4,5 & 6), corroborating with prior reports of detection of additional CNVs in ~ 30% of cases [30]. In one of these individuals this was secondary to an inversion leading to a unique chromosomal rearrangement with two deletions: 579 kb in 22q11.1 and 7.2 Mb in 22q13.3 (Fig. 1). Furthermore, the detection of PMS individuals harboring ring chromosome 22 is essential, as these individuals have an increased risk of cancer, due to possible loss of the NF2 gene (2–4%) [29, 31, 32]. The two individuals reported herein continue follow up, without NF-2 related symptoms. Last, a single family harbored a parental balanced translocation causing PMS in the proband (t(10;22)(p15.3;q13.31)).

Three participants harbored the same recurring pathogenic variant: p.Ala1302GlyfsTer69 in *SHANK3* (individuals 2, 8, and 16). This recurring variant occurs within a homopolymer run of 8 guanines, and has been previously reported as a recurring variant among PMS individuals with variable phenotypic features [6, 9, 23–33]. Given the complexity of sequencing and multiple transcripts, this variant has also been designated alternate names, which complicates consistency in variant reporting (rs797044936). Possible mechanisms for this recurring variation include the vulnerability of homopolymer stretches (X10) along with epigenetic alterations in chromatin folding [34–35]. Another interesting finding is the paternally inherited variant in participant 17 (p.L510fsTer32). While the proband was diagnosed with ASD and mild ID (DQ85), his father had ADHD without additional manifestations. In the father, this variant was de novo. Truncating variants within *SHANK3* are thought to be fully penetrant, and although inherited truncating variants have been reported, recent reanalysis of these variants revealed that they are relatively common in healthy population databases [6]. This may implicate noncausality, but the p.L510fsTer32 variant is absent from population databases (GnomAD V4 = 0) and is predicted to lead to early termination of RNA translation and nonsense-mediated decay (NMD). Thus, we classified this variant as likely pathogenic. Mechanisms that may explain the mild paternal phenotypic expression include paternal mosaicism (although the blood DNA allele ratio was not suggestive for this option) along with additional multifactorial determinants [36]. Given the wide phenotypic variability along with the core feature of ASD in the proband and the mechanism of the LOF variant within *SHANK3*, we consider this variant as PMS causing.

Regression and loss of acquired skills is a recurring feature of PMS (reported in 40–70% of persons) [7, 30, 31, 37]. Early childhood regression was noted in 10 participants (59%), whereas two were reported to have undergone multiple regression episodes. Autoimmune encephalitis (AIE) has been suggested as a possible cause [38], with improvement under treatment with immune modulation. Thus, workup has been recommended for individuals with abrupt onset, severe course and/or treatment failure [31]. As the two individuals reported herein were assessed later in our clinic, evaluation for possible AIE was not conducted.

Lymphedema was present in four individuals (23%), all harboring CNVs encompassing the *CELSR1* gene. This finding is in line with prior reports implicating this gene in the pathogenesis of lymphedema formation [30]. Finally, in accordance with a reported dual diagnosis of genetic conditions among ~ 5% of individuals [39–40], one of our participants (5.6%) had a dual diagnosis of PMS and long QT. Since patients harboring CNV’s with phenotypic consistency to PMS did not proceed to NGS analysis in most cases, we cannot entirely exclude the possibility of individuals with additional genetic conditions that were not detected.

Prenatal findings have been reported in a minority of PMS individuals, including IUGR, single umbilical artery dysplasia, cystic kidney dysplasia, and complex congenital heart disease, among additional features [15, 31, 41–44]. In our cohort, abnormal prenatal ultrasonographic findings were noted in 3 individuals (18%), all harboring CNVs. Two fetuses presented with mild bilateral hydronephrosis, and the other fetus had microcephaly. Thus, PMS should be considered in the differential diagnosis of hydronephrosis in the prenatal setting, even as an isolated finding. Notably, in most participants, prenatal ultrasonographic surveillance was normal.

Current therapeutic options for PMS are symptomatic and nonspecific. The complexity in the treatment of PMS patients is related to multisystem involvement and a lack of measurable parameters for assessing treatment efficacy. While the use of atypical antipsychotics, phenothiazines and stimulants has been recommended for PMS patients [7, 12, 44], in our experience these were not well tolerated, and all were discontinued owing to side effects, including catatonia, aggressiveness, anxiety, mood lability or lack of efficacy.

Treatment trials of oxytocin, recombinant growth hormone (rGH) and insulin-like growth factor 1 (IGF1) have been conducted for PMS, with promising results for the latter two [45–48]. One participant was treated with IGF1 as part of a compassionate use protocol and discontinued after two months without clear beneficial effects. As further aspects of treatment with rGH are being investigated [48] and additional treatment modalities, such as exosome therapy [49], may be available, a referral clinic is key as a platform for recruitment to clinical trials.

Cannabis agents have been explored for their potential therapeutic effects in children with ASD or epilepsy [50–52]. However, data regarding its use in treating specific conditions such as PMS are lacking, and individual responses can vary widely. Both THC (tetrahydrocannabinol) and CBD (cannabidiol) affect the endocannabinoid system, which plays a role in regulating mood, behavior, pain, and other physiological functions [53]. Preliminary research suggests that CBD, a nonpsychoactive compound in cannabis, may have calming effects and influence anxiety, agitation and sleep [50–57].

Eight participants in the cohort received CBD, and 7 caregivers reported subjective improvement in overall behavior and a reduction in seizure frequency within one month of treatment. Parents also mentioned increased daytime tranquility, with a decrease in anxiety, tantrums, impulsive behavior, violence, and improved night sleep. Whereas the appropriate dosage and formulation are not determined, a common practice is to commence with low doses and monitor for side effects [50]. As research on the potential therapeutic indications of cannabis-derived CBD products is ongoing, more rigorous studies in patient populations with rare disorders such as PMS, are warranted and offer a promising treatment avenue.

Interestingly, 3 individuals with PMS from our cohort (two were diagnosed with ASD and one had severe ID and his communication assessment was difficult) had siblings who were also diagnosed with ASD. Nevertheless, the sibling did not harbor the *SHANK3* pathogenic variation. Genetic workup within these families, including CMA and trio ES, did not reveal a molecular diagnosis for the affected sibling. Since ASD is recognized in most but not all PMS patients without a clear genotype–phenotype correlation [18], additional genetic and/or environmental factors probably affect ASD susceptibility in these families and warrant further investigation.

The major limitation of the study is the small sample size. Despite our extensive recruitment process, it is likely that not all individuals diagnosed with PMS in Israel were enrolled. For example, two sisters diagnosed with schizophrenia and ID harboring SHANK*3* variations were reported by Alkelai et al. [58] but were not assessed at our clinic; hence, they were not included in our cohort. We hope that in the future, additional individuals will be enrolled in the registry. In addition, this was a retrospective cross-sectional study, and uniform long-term follow-up in the same clinic is ongoing. Most individuals were evaluated as children or adolescents, and only 2 were adults. Thus, our study does not cover adult-onset features and characteristics of PMS, such as psychiatric, behavioral, and other factors, or life expectancy.

To conclude, we present the first Israeli cohort of individuals diagnosed with PMS, in which all participants were assessed in the clinic by a single provider. We consider such databases and specialized clinics for patients with rare diseases to be highly desirable. These factors enable the medical team to gain expertise in many aspects of the condition, improve outcomes, and deepen the understanding of the natural history of the disease. Furthermore, given the expected advances in treatment opportunities, this PMS clinic will hopefully enable access to clinical trials and care opportunities, leading to improvements in care for individuals diagnosed with PMS in Israel and worldwide.

## Data Availability

The datasets used and/or analyzed during the current study are available from the corresponding author on reasonable request.
